# Efficacy of dialysis for the treatment of patients with diabetic nephropathy: Study protocol

**DOI:** 10.1097/MD.0000000000017923

**Published:** 2019-11-22

**Authors:** De-rong Shao, Yue Zhou

**Affiliations:** a Department of Nephrology; b Department of Endocrinology, The Second Affiliated Hospital of Mudanjiang Medical University, Mudanjiang, China.

**Keywords:** diabetic nephropathy, dialysis, efficacy, safety

## Abstract

**Background:**

This study aims to assess the efficacy and safety of dialysis for the treatment of patients with diabetic nephropathy (DN).

**Methods:**

We will comprehensively retrieve the following databases of Cochrane Library, PUBMED, EMBASE, Global health, CINAHL, PsycINFO, Scopus, CBM, Wangfang, and CNKI for studies related to the topic. We will search all those electronic databases from their inceptions to the present without restrictions of language and publication status. Two authors will independently conduct all procedures of study selection, data collection, and risk of bias assessment. We will apply RevMan 5.3 software for statistical analysis.

**Results:**

We will systematically investigate the efficacy and safety of dialysis for DN through assessing primary and secondary outcomes. The primary outcomes include improvement in renal function, as assessed by the urinary albumin/creatinine ratio, estimated glomerular filtration rate, and serum creatinine levels. The secondary outcomes consist of levels of inflammatory markers, endothelial dysfunction markers, quality of life, and any expected and unexpected adverse events.

**Conclusion:**

This study will present evidence on the efficacy and safety of dialysis for the treatment of patients with DN.

**Systematic review registration:**

PROSPERO CRD42019149699.

## 1 Introduction

Diabetic nephropathy (DN) is one of the most common and server complications in patients with diabetes mellitus (DM); and it occurs in 20% to 40% of all DM patients.^[1–5]^ It has become a major cause of end-stage renal disease (ESRD), and accounts for over 40% ESRD.^[6,7]^ It is often manifested as swollen limbs, darker urine, shortness of breath, fatigue, nausea or vomiting, and a metallic taste in month.^[8–10]^ It is associated with renal dysfunction and a high risk of cardiovascular death at the later stages.^[11–13]^ Thus, it is very important and crucial to manage DN at the early stage to prevent its further progress and possible complications.^[14,15]^

Dialysis has been reported for the management of patients with DN effectively.^[16–20]^ However, no systematic review has investigated the efficacy and safety of dialysis for DN. Therefore, this study will be undertaken to assess the efficacy and safety of dialysis for patients with DN.

## 2 Methods

### 2.1 Dissemination and ethics

The results of this study will be published at peer-reviewed journals. Ethical approval is not required, because all data we will use in this study has been published.

### 2.2 Criteria for considering studies for inclusion

#### 2.2.1 Types of studies

This study will only consider randomized controlled trials (RCTs) of dialysis for the treatment of patients with DN for inclusion. However, any other studies, such as animal studies, reviews, case studies, non-controlled studies, and quasi-RCTs will be excluded.

#### 2.2.2 Types of participants

Patients diagnosed with DP, who meet all eligible criteria, will be included in this study without any restrictions of ethnicity, sex, and age.

#### 2.2.3 Types of interventions

All patients in the experimental group must receive dialysis treatment.

Patients in the control group can be treated with any interventions, but not the dialysis treatment.

#### 2.2.4 Type of outcome measurements

The primary outcomes include improvement in renal function, as assessed by the urinary albumin/creatinine ratio, estimated glomerular filtration rate, and serum creatinine levels. The secondary outcomes consist of levels of inflammatory markers, endothelial dysfunction markers, quality of life, as measured by the 36-Item Short Form Health Survey, and any expected and unexpected adverse events.

### 2.3 Search methods for the identification of studies

#### 2.3.1 Electronic database records searches

We will comprehensively search Cochrane Library, PUBMED, EMBASE, Global health, CINAHL, PsycINFO, Scopus, CBM, Wangfang, and CNKI for studies on assessing the efficacy and safety of dialysis for the treatment of DN. All electronic databases will be searched from the inceptions to the present. There will be no limitations on the language, time of publication, and publication status. The specific search strategy for Cochrane Library is presented in Table 1. We will also adapt similar search strategies to the other electronic databases.

**Table 1:**
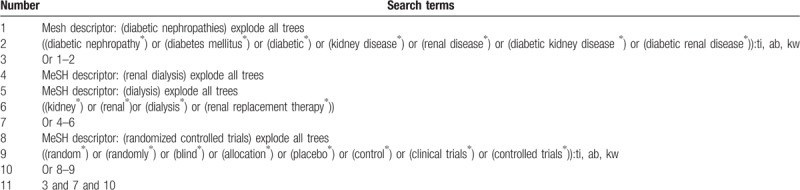
Search strategy for Cochrane Library.

#### 2.3.2 Searching other records source

To avoid missing any other relevant studies, we will also search other source records, including conference proceedings, clinical registry, and reference list of relevant reviews.

### 2.4 Data collection and analysis

#### 2.4.1 Study selection

After the literature records search, 2 authors will independently scan studies for eligibility via reading the titles and abstracts of all those sources. All irrelevant sources will be excluded after the initial selection. Then, the full-texts of the remaining studies will be read carefully to judge if they meet all inclusion criteria, and we will exclude all unqualified studies with specific reasons at different stages. If there are different opinions regarding the study selection between 2 authors, we will invite another author to solve them through discussion.

#### 2.4.2 Data extraction

We will extract the following data from each qualified study: title, primary author, time of publication, country, ethnicity, age, sex, diagnostic criteria, inclusion and exclusion criteria, sample size, randomization methods, blinding, concealment, regimens of treatments and comparators, outcome details, and adverse events. Two authors will independently carry out data extraction according to the pre-designed form of data extraction. Any discrepancies regarding the data extraction between 2 authors will be solved by a third author if it is necessary.

#### 2.4.3 Methodological quality assessment

The methodological quality of selected studies will be appraised using Cochrane Handbook for Systematic Reviews of Interventions tool, which evaluate factors, comprising of 7 domains. Each domain is further assessed as high, unclear, or low risk of bias. Two authors will independently evaluate the methodological quality for each qualified study. Any divergences between the 2 authors will be solved by a third author through discussion.

#### 2.4.4 Dealing with missing data

If any missing data occurs, we will contact original authors to request it. If we cannot obtain such data, we will analyze the current available data and will explore its effects in the discussion section.

#### 2.4.5 Data synthesis

In this study, we will apply RevMan 5.3 software for statistical analysis. Risk ratio and 95% confidence intervals (CIs) will be collected for enumeration data, while mean difference or standardized mean difference and 95% CIs will be utilized to calculate continuous outcome data. The *I*^2^ statistic will be utilized to determine the heterogeneity among the qualified studies. When *I*^2^ ≤ 50%, acceptable heterogeneity is considered, otherwise, when *I*^2^ > 50%, high heterogeneity is regarded. We will use a fixed-effects model to pool the data if *I*^2^ ≤ 50%. Under such situation, if >2 included studies on the same interventions and outcomes, we will conduct meta-analysis to analyze the pooled data. On the other hand, we will utilize a random-effects model to synthesize the data if *I*^2^ > 50%. At the same time, we will perform subgroup analysis and meta-regression test to explore the potential factors that cause the significant heterogeneity among qualified studies.

#### 2.4.6 Reporting bias

We will conduct Funnel plot and Egger regression test to check if there is any reporting bias when >10 qualified studies are included.^[21]^

#### 2.4.7 Subgroup analysis

We will investigate the source of heterogeneity using subgroup analysis based on different interventions, controls, and outcomes.

#### 2.4.8 Sensitivity analysis

We will carry out sensitivity analysis to investigate the robustness and stability of outcome results by removing low methodological quality studies.

## 3 Discussion

Although a variety of studies have reported the dialysis for the treatment of patients with DN, its efficacy and safety is still inconsistent. Thus, this study will systematically evaluate the efficacy and safety of dialysis for the treatment of patients with DN. This study will provide helpful evidence of dialysis for treating DN at evidence-based medicine levels. The findings of this study will also affect the clinical practice and health related policy to improve DN treatment approach.

## Author contributions

**Conceptualization:** De-rong Shao, Yue Zhou.

**Data curation:** De-rong Shao, Yue Zhou.

**Formal analysis:** De-rong Shao, Yue Zhou.

**Investigation:** Yue Zhou.

**Methodology:** De-rong Shao.

**Project administration:** Yue Zhou.

**Resources:** De-rong Shao, Yue Zhou.

**Software:** De-rong Shao.

**Supervision:** Yue Zhou.

**Validation:** De-rong Shao, Yue Zhou.

**Visualization:** De-rong Shao, Yue Zhou.

**Writing – original draft:** De-rong Shao, Yue Zhou.

**Writing – review & editing:** De-rong Shao, Yue Zhou.
